# Tumoral Calcinosis of the Cervical Spine Associated with a Pathologic Odontoid Fracture

**DOI:** 10.1155/2022/2798490

**Published:** 2022-01-07

**Authors:** Andy Y. Wang, Joseph N. Tingen, Eric J. Mahoney, Ron I. Riesenburger

**Affiliations:** ^1^Department of Neurosurgery, Tufts Medical Center, Boston, MA, USA; ^2^Department of Surgery, Tufts Medical Center, Boston, MA, USA

## Abstract

Tumoral calcinosis involves focal calcium deposits in the soft tissues surrounding a joint and most commonly occurs in the hips and elbows, rarely in the cervical spine. Furthermore, it has not been known to be associated with pathologic fractures. To the best of our knowledge, our case report highlights the first case of a pathologic type II odontoid fracture associated with adjacent tumoral calcinosis, resulting in pain, dysphagia, and severe spinal stenosis. The patient underwent a posterior occipitocervical fusion and C1 laminectomy, along with planned tracheostomy and gastrostomy to avoid expected difficulty with postoperative extubation and dysphagia. Additionally, we present a review of existing literature on tumoral calcinosis in the upper cervical spine.

## 1. Introduction

Tumoral calcinosis is a rare clinical disease typified by focal and/or multifocal calcium masses in the soft tissue surrounding a joint in the body, typically in the juxta-articular regions [1, 2]. The first cases of calcifying masses were presented by Giard in 1898 and Duret in 1899, where they found deposition in the connective tissue surrounding a joint [3, 4]. Subsequently, the term “tumoral calcinosis” was first used by Inclan et al. in 1943, characterizing large juxta-articular lobular calcified masses showing multiple calcium-filled cysts [5]. There are several similar differentials to consider such as calcific tendonitis and calcific myonecrosis, for which imaging can aid in distinguishing [2]. Tumoral calcinosis has been found to mostly affect patients in the first two decades of life, especially in populations of African descent and without sex predominance [6]. Most of the depositions occur surrounding larger joints, including, in descending order, the hip, elbow, shoulder, foot, and wrist [6]. Tumoral calcinosis in the cervical spine is especially rare, with only a handful of reported cases.

In this case presentation, we report the first known instance of tumoral calcinosis in the cervical spine associated with a pathologic type II odontoid fracture. Typically, tumoral calcinosis patients have shown a lack of osseous destruction. However, this case demonstrates the possible association of tumoral calcinosis in the cervical spine with pathologic fracture, highlighting the need for increased awareness of tumoral calcinosis involvement in the cervical spine and the possibly increased risk of cervical instability in such patients predisposed to the disease.

## 2. Case Presentation

A 73-year-old female presented to our neurosurgery clinic with cervical pain. The patient uses a walker but did not remember any obvious fall or injury mechanism and presented 3 weeks prior to an outside clinic with moderate aching pain at her midposterior neck. The pain was relieved with ibuprofen, with no radiation, numbness, tingling, burning, or paresthesias. The patient did have dysphagia supported by swallow studies from an outside institution, but no decreased appetite nor dyspnea. Imaging completed at that time showed a type II odontoid fracture, and she was placed in a hard cervical collar. Past medical history is notable for tumoral calcinosis in the shoulder, hyperparathyroidism, Parkinson's disease, difficult intubation, essential hypertension, and osteoarthritis. Past surgical history is notable for multiple elbow surgeries for tumoral calcinosis. Physical exam was limited in the upper extremities with her history of shoulder surgeries, though patellar reflexes were 2+ bilaterally and negative for Babinski sign and ankle clonus.

**Figure 1:**
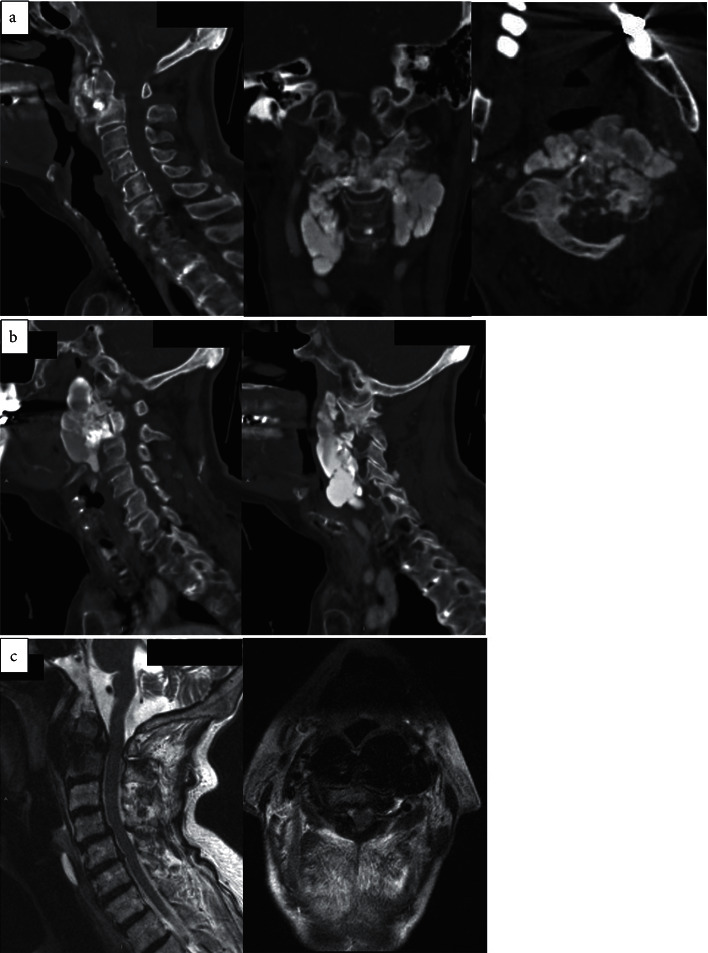
Noncontrast CT scans and T2-weighted MRI of preoperative odontoid fracture with associated tumoral calcinosis. (a) Sagittal, coronal, and axial CT images, respectively from left to right, show pathological type II odontoid fracture, (b) left: left parasagittal CT image shows extensive tumoral calcinosis; right: right parasagittal CT image shows extensive tumoral calcinosis, and (c) left: T2-weighted sagittal MRI shows spinal cord compression at C1-C2; right: T2-weighted axial MRI at C1-C2.

Noncontrast computed tomography (CT) of the head and neck revealed an unstable type II odontoid fracture with significant anterolisthesis of the odontoid over C2 (Figure 1(a)). The left and right parasagittal CT images confirmed a large amount of abnormal bone ventral to C2 consistent with tumoral calcinosis which displaces the pharynx (Figure 1(b)). Magnetic resonance imaging (MRI) of the cervical spine showed C1-2 subluxation with the anteriorly displaced posterior arch of C1 resulting in severe stenosis (Figure 1(c)).

Based on the abovementioned findings along with the patient's history of tumoral calcinosis in the shoulder, we determined the diagnosis of pathological type II odontoid fracture secondary to tumoral calcinosis with C1 stenosis and cervical instability resulting in pain and dysphagia. The patient opted for surgical intervention and underwent a posterior occipitocervical fusion (occiput to C3) with posterior C1 laminectomy. She also underwent planned tracheostomy and G-tube placement under the same anesthetic to avoid likely difficulties with postoperative extubation and dysphagia.

**Figure 2:**
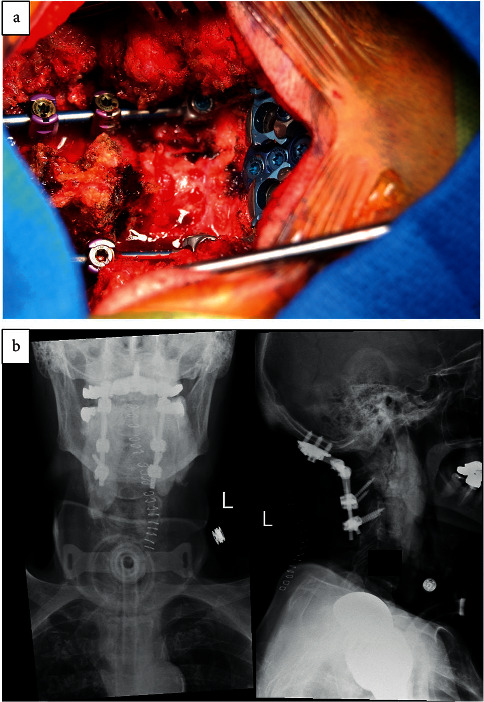
(a) Intraoperative photo of the posterior occipitocervical (occiput to C3) fusion with a posterior C1 laminectomy. (b) Postoperative AP and lateral x-rays of the cervical spine showing the construct.

The patient was brought to the operating room and intubated under general anesthesia with placement of a Foley catheter and neuromonitoring leads to monitor for intact motor and somatosensory evoked potentials. Upon exposure, the C1-C2 area appeared very unstable. The occipital keel plate was placed, and three midline keel screws were placed with bilateral purchase. Bilateral C2 pars screws and bilateral C3 lateral mass screws were placed, and rods were placed to stabilize the occipitocervical junction (Figure 2). For the decompression, a wide C1 laminectomy was performed. The thecal sac appeared well decompressed. Locally harvested autograft was denuded of soft tissue and combined with osteomatrix bone graft extender and laid along the posterior decorticated posterolateral elements to complete the arthrodesis. Final somatosensory evoked potentials, and motor evoked potentials were stable and intact.

Subsequently, general surgery performed the tracheostomy and Stamm gastrostomy under the same anesthetic. During the tracheostomy, it was noticed that her tracheal rings were calcified. Attempt at percutaneous G-tube placement failed, as the mass effect from the tumoral calcinosis posterior to the pharynx prevented the passage of the endoscope. Therefore, an open G-tube was placed. The patient tolerated the procedure well and was discharged to rehab. Postoperative visits showed well-positioned hardware, and the tracheostomy was discontinued without event after 2 weeks. Her swallowing also improved slowly, and the G-tube was removed three months after surgery. She reports doing well 6 months following surgery with improvement in her neck pain.

## 3. Discussion

Tumoral calcinosis is characterized by calcium deposition in the juxta-articular regions and is known to occur mostly in regions outside of the spine such as the hip and elbow. Calcification in the spine is much less common since it consists of much smaller joints, and tumoral calcinosis in the cervical spine is rarer. The patient described in this case report was found to have a pathological type II odontoid fracture secondary to tumoral calcinosis with C1 stenosis and cervical instability. Smack et al.'s classification of tumoral calcinosis separated the disease into three subtypes—normophosphatemic primary, hyperphosphatemic primary, and secondary tumoral calcinosis [7]. Due to our patient's previous history with hyperparathyroidism, her ultimate diagnosis was secondary tumoral calcinosis. However, unlike any other reported cases of tumoral calcinosis in the cervical spine, our patient's cervical instability is associated with a pathological fracture. This case report raises the concern for pathologic fractures to be considered when determining the diagnosis and management of tumoral calcinosis.

We considered several differential diagnoses (Table 1). In terms of the other calcifying disorders, calcific tendinitis shows increased calcium levels centered within a tendon but does not involve deposition of calcium into a mass. Synovial osteochondromatosis involves calcification in the sheath between joints and tendons but occurs in an intra-articular location rather than periarticular like in tumoral calcinosis. Other differential diagnoses such as myositis ossificans, heterotopic ossification, and tophaceous gout are characterized by nonlobular masses [2, 6]. Imaging is particularly helpful to help differentiate findings specific to tumoral calcinosis. On radiographs, tumoral calcinosis has distinguishable features in the periarticular region, including amorphous, cystic, and multilobulated calcifications [6]. These masses can be better distinguished by CT scans, particularly showing fluid-fluid levels due to calcium layering in a process termed the sedimentation sign. The CT scans in Figure 1 include findings of cystic loculi and calcium layering, aiding in our diagnosis of tumoral calcinosis. The primary treatment for tumoral calcinosis in all joint regions is surgical excision of the calcified mass [6]. Since recurrence is common, some have attempted to use low phosphate diets or phosphate binders.

Compared to other case reports of tumoral calcinosis in the cervical spine, this patient's associated pathologic fracture with tumoral calcinosis is singly unique. Only 22 reported cases involve tumoral calcinosis around C1 or C2, and none of these cases have been associated with a pathological fracture (Table 2). Many of the cases in the upper cervical spine were also presented with spinal stenosis and dysphagia [8–19]; however, tumoral calcinosis and anterolisthesis resulting in a fracture of the cervical joint are essentially absent from the literature. This case report provides a valuable addition to the currently limited available literature. The current literature tends to focus on the pathological etiology and how tumoral calcinosis has been associated with chronic renal failure, systemic sclerosis, and collagen and vascular disorders. We present a new perspective, emphasizing a new risk that may emerge from tumoral calcinosis. Although a pathological fracture secondary to tumoral calcinosis in the cervical spine does not appear in the literature, Miura et al. presented a case of tumoral calcinosis in the thoracic spine with an associated fracture that parallels our patient's condition [20]. It is imperative that these factors are considered in the possible association of fracture risk with tumoral calcinosis. Our current patient also presents with Parkinson's disease, not described as a comorbidity with the other case reports. Patients with compromised motor control may be at further risk of spinal cord instability and injury, which may necessitate further precautions in preventing a joint fracture.

To our knowledge, this is the first report of tumoral calcinosis in the cervical spine with an associated pathological type II odontoid fracture. This case raises the possibility of tumoral calcinosis involving surrounding regions, which may apply to tumoral calcinosis outside of the spine as well. Further research is needed to better understand the biochemical underpinnings of this process, such as the role of inflammatory markers [20]. Physicians and clinicians should be wary of the possibility of pathologic fracture in patients with tumoral calcinosis.

## Figures and Tables

**Table 1:** Differential diagnoses considered.

Differential diagnosis	Rationale
Infection	No local (redness, swelling, and other skin findings) nor systemic signs (fever) and no disc space involvement
Type I odontoid fracture secondary to trauma	Type I involves avulsion of the rostral tip of the odontoid process; in this case, the fracture is at the base
Type III odontoid fracture secondary to trauma	Type III involves fracture through the body of C2; in this case, the fracture is at the base
Type II odontoid fracture secondary to trauma alone	Type II involves fracture through the base of the odontoid process, which matches this case, but there is no identifiable fall or injury mechanism
Pathologic type II odontoid fracture secondary to primary bone tumor or bone metastases	No evidence of primary bone tumor or metastases on imaging, no clinical symptoms such as weight loss, and no personal or family history
Pathologic type II odontoid fracture secondary to tumoral calcinosis	This differential matches the type of odontoid fracture and explains why there is a fracture despite no obvious injury mechanism. The patient has a history of tumoral calcinosis and along with the radiological findings, differentiates tumoral calcinosis from other calcifying conditions

**Table 2:** Existing case reports of tumoral calcinosis involving the upper cervical spine at C1 or C2.

Authors, year	Sex	Age	Location	Associated conditions	Clinical presentation	Treatment
Kokubun et al., 1996	F	68	C1-C2	NP	Neck pain below the occiput	Resection, laminectomy
Arginteanu et al., 1997 [9]	F	65	C1–C5	SS	Neck pain	Decompression, resection, fusion
Mooney et al., 1997	M	17 mo.	C1-C2	NP	Torticollis	Resection, limited laminectomy
Ward et al., 1997	F	62	C1-C2	SS	Left-side neck pain, limited range of motion, tenderness	CT-guided aspiration
Durant et al., 2001 [8]	M	78	C1-C2	OA, HTN	Pannus from RA	Posterior fusion, excision
Matsukado et al., 2001 [1]	F	54	C2–C4	RF, RM	Cervical pain, weakness	C-2 laminectomy, C3-4 laminoplasty
Van de Perre et al., 2003	F	74	C2–C7	SS	Increasing joint/neck pain	NS
Olsen et al., 2004	F	75	C2–C4	SS	Torticollis, swelling	CT-guided biopsy and excision
Smucker et al., 2006 [11]	M	60	C1-C2	SS	Severe upper neck pain, myelopathy	Decompression, resection, fusion
Smucker et al., 2006 [11]	F	59	C2–C5	SS	Neck pain	Laminectomy, resection, fusion
Tuy et al., 2008	F	50	C2-C3	RF, previous scleroderma	Neck pain	Resection
Shoji et al., 2012	F	34	C1-C2	SS	Severe headache, narrowed face	NS
Bisson-Vaivre et al., 2013	F	72	C1-C2	SS	Severe upper neck pain	Colchicine treatment
Chang et al., 2013	F	44	C1-C2	RF, HTN	Severe neck soreness, headache	C1 laminectomy, fixation, fusion
Daumas et al., 2013	F	60	C2–C5	SS	NS	NS
Lebl et al., 2013	F	63	C1-C2	SS	Severe neck pain, limited range of motion	Physical therapy, steroid injections
Al-Khudairi et al., 2015 [16]	F	62	C2–C6	SS	Weakness, altered sensation, inability to mobilize	Decompression, fusion
Ashraf et al., 2015	M	5 mo.	C2-C3	Familial RA	Torticollis	CT-guided biopsy, excision
Fatehi et al., 2016	F	73	C2-C3	HTN, RF	Growing painful mass	Only hemodialysis
Mooney et al., 2017 [18]	F	60	C1-C2	HTN	Dysphagia, neck pain	Transoral decompression
Ebot et al., 2019	F	71	C1-C2	NS	Neck pain	C1-C2 laminectomy
Steward et al., 2019	F	4 mo.	C1-C2	NS	Loss of head control, neck hypotonia	Surgical excision
Current case report	F	73	C1-C2	HTN, Parkinson's, HP	Achy neck pain, dysphagia, pathological fracture	Occ-cervical fusion, C1 laminectomy, tracheostomy

M: male, F: female, HP: hyperparathyroidism, OA: osteoarthritis, NP: no particular cause, NS: not specified, HTN: hypertension, RA; rheumatoid arthritis, RF: renal failure, RM: radiculomyelopathy, SS: systemic sclerosis/scleroderma, RI: renal insufficiency.

## Data Availability

Patient information was accessed through medical records at Tufts Medical Center and is unavailable for release due to patient confidentiality.
